# Cartap Poisoning with Paroxysmal Supraventricular Tachycardia: A Case Report

**DOI:** 10.31729/jnma.8772

**Published:** 2024-10-31

**Authors:** Newton Ashish Shah, Bibek Rajbhandari, Santosh Banstola, Manish Acharya, Rupesh Joshi, Shree Krishna Luitel, Manish Yadav

**Affiliations:** 1 Institute of Medicine, Tribhuvan University Teaching Hospital, Maharajgunj, Kathmandu, Nepal; 2 Department of Emergency Medicine, Tribhuvan University Teaching Hospital, Maharajgunj, Kathmandu, Nepal

**Keywords:** *insecticides*, *nereistoxin*, *poisoning*, *thiocarbamates*

## Abstract

Cartap, a nereistoxin derivative from the marine annelid Lumbriconeresis heteropoda, is widely used as a pesticide, targeting pests like caterpillars. While the WHO classifies it as moderately hazardous, with a recommended daily intake of 0.05 mg/kg, human toxicity reports are limited. A 34-year-old female was admitted after consuming an unknown poison. She reported epigastric pain, burning sensations, sweating, vomiting, dyspnea, palpitations, and restlessness, compounded by alcohol intake. Initially treated for organophosphate poisoning, her cholinesterase level was 8.91. It was later confirmed she ingested 100 ml of 50% concentration cartap. Treatment included amiodarone for supraventricular tachycardia and intravenous N-acetylcysteine, magnesium sulfate, and midazolam for general tonic clonic seizure. Cartap, toxic through ingestion and skin contact, can cause symptoms such as vomiting, convulsions, and cardiac issues like supraventricular tachycardia. Supportive care is crucial, and awareness of its risks is necessary.

## INTRODUCTION

Cartap, an analogy of nereistoxin derived from marine annelid Lumbriconeresis herteropoda, is a commonly used pesticide used to control pests and insects like caterpillar.^1^ Its fundamental chemical structure is S, S-[2-(dimethylamino)-1,3-propanediyl] dicarbamothioate, commonly employed as the hydrochloride (cartap hydrochloride). According to the WHO, it is moderately hazardous with a maximum acceptable daily intake of 0.05mg/kg.^2^ Cartap was used in Japan in 1967 AD.^3^ Although reported as less hazardous, many fatal cases have been reported.^4^ Despite its wide usage, few reports on human exposure to cartap resulting in clinical toxicology exist.

## CASE REPORT

A 34-year-old female presented after deliberately ingesting an unknown substance 30 minutes prior. The patient complained of burning epigastric pain, increased sweating, and excessive secretions. She could not recall the amount and nature of the substance ingested. She experienced multiple episodes of non-bloody, non-bilious, and non-projectile vomiting. Additionally, she had shortness of breath, palpitations, and restlessness following consumption of the substance. Gastric lavage with soap water and self induced vomiting was attempeted but As the patient deteriorated, she was referred to our center. On presentation. The patient appeared restless with above sign and symptoms and spoke incoherently. She also displayed one episode of abnormal body movements, including eye rolling and post-ictal confusion, which indicated that her level of consciousness was impaired. The patient had no other comorbidities but had been consuming alcohol for the past five months with last intake of one day prior and had irregular menstrual cycles. There was also a history of suicidal attempts over the past nine months.

On examination, her Glasgow Coma Scale (GCS) was 8/15 (E2V1M5); the low Glasgow Coma Scale (GCS) score was attributed to the postictal phase, but it returned to normal after approximately 30 minutes. Her vitals revealed a respiratory rate of 24 per minute, blood pressure of 100/70 mmHg, and a pulse rate of 200 beats per minute, indicating marked tachycardia.

**Figure 1:**
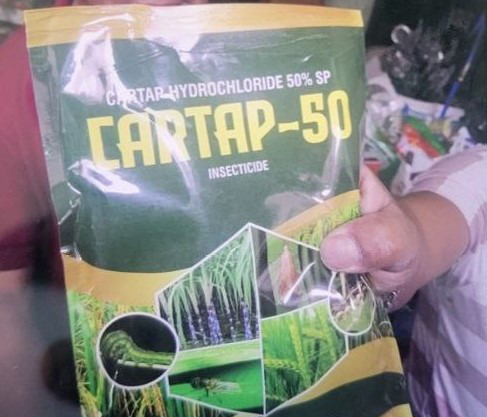
Cartap hydrochloride packet retrieved by relative

SpO2 was 98% with a facemask at 6 liters per minute. On auscultation, crepitations were heard at the base of the axilla. Pupils were reactive with horizontal nystagmus. The abdomen was soft and non-tender with no organomegaly. At first, she was managed as organophosphate poisoning. But upon investigations, her cholinesterase level was only 8.91 units/mL and the relatives later brought the packet and reported ingesting approximately 100 ml of Cartap (50%), amounting to about 15 grams.(Figure 1)

When the blood investigations were conducted, along with ABG analysis that revealed (High Anion Gap Metabolic Acidosis) HAGMA (pH - 7.076, HCO3 - 2.6 mmol/L); Intravenous sodium bicarbonate was administered according to the calculated deficit. Following the initial correction, arterial blood gas (ABG) analysis showed that the parameters had returned to optimal levels, fluid resuscitation was initiated along with intravenous administration of N-acetylcysteine (NAC) 9g over 50 minutes, magnesium sulphate (MgSO4) 2g.

**Table 1:** Blood investigations

Test	Value	Reference Range
TLC	12400	4000-11000/cumm
Hb	15.2	12.0-16.0 mg/dL
Platelets	126000*	150000-450000/cumm
Na	145	135-145 mEq/L
K	3.3	3.5-5.2mEq/L
Total bilirubin	25	5-21 umol/L
Direct bilirubin	7	<4 uMol/l
ALT	107	0-35U/L
AST	183	0-35U/L
PT/INR	18.8/1.43	
Urea	5.2	2.8-7.2mmol/L
Creatinine	69	74-110uMol/L
TSH	0.567	0.35-4.94 uIU/mL
T4	11.37	9-19 pmol/L
T3	2.83	2.4-6.0pmol/L
Mg	2.1	1.7-2.5 mg/dL
Total Cholesterol	4.4	3.5-5.1 mmol/L
High-Densiity Lipid(direct)	0.9	0.8-1.6 mmol/L
Low-Density Lipid	3.0	<4.0 mmol/L
Triacylglycerol	1.2	0.5-1.8 mmol/L
HbA1c	9.8	4.5-6.4%
Cholinestare level	8.91	3.93-10.8 KU/L
Troponin	Negative	
PBS	Normocytic normochromic RBC, with normal WBC without any atypical cells	
Vitamin B12	540	
Vitamin B6	6.9	
Iron	76	60-130 Ug/dl
Ferritin	256	4.6/204 ng/dL
TIBC	248	200-400Ug/dL

**Figure 2:**
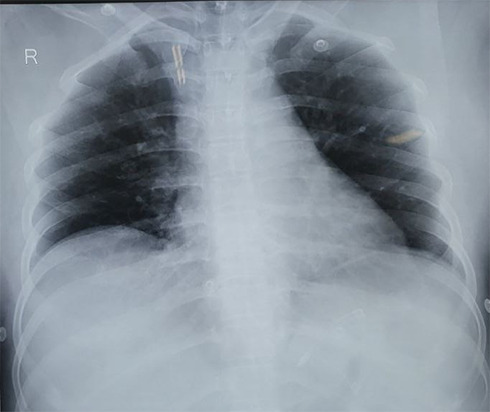
Xray showing bilateral basal opacities

Electrocardiogram (ECG) showed persistent Supraventricular Tachycardia (SVT) likely Atrioventricular Nodal Reentrant Tachycardia (AVNRT) for which adenosine was used but did not control the rate. An amiodarone infusion was then administered, resulting in successful cardioversion. She was managed with midazolam for Generalised Tonic Clonic Seizure (GTCS ). An echocardiogram (ECHO) revealed normal findings, with left ventricular ejection fraction (LVEF) being 60%. Abdominal ultrasound (USG) was normal. A chest X-ray showed alveolointerstitial infiltrates ( Figure 2), and a 24-hour Holter study later revealed a single atrial premature beat (APB) and a supraventricular couplet. No sustained SVT, ventricular premature contractions (VPC), ventricular tachycardia (VT), or ventricular fibrillation (VF) were noted. There were no significant sinus pauses or atrioventricular (AV) block.

The investigation revealed several abnormal laboratory findings. The complete blood count showed a slightly elevated total leukocyte count (TLC) of 12,400/cumm (reference: 4,000-11,000/cumm) and reduced platelet count of 126,000/cumm (reference: 150,000-450,000/cumm), indicating a possible inflammatory response. Electrolyte levels showed a slightly low potassium (K) of 3.3 mEq/L (reference: 3.5-5.2 mEq/L). Liver function tests revealed significantly elevated levels of ALT (107 U/L) and AST (183 U/L) (reference: 0-35 U/L) along with a total bilirubin level of 25 umol/L (reference: 5-21 umol/L) and direct bilirubin of 7 umol/L (reference: <4 umol/L), suggesting hepatocellular injury, possibly due to toxin exposure. Coagulation tests indicated prolonged prothrombin time (PT) of 18.8 seconds and an INR of 1.43, which may point to liver dysfunction or coagulation impairment. Kidney function appeared normal, with creatinine at 69 uMol/L (reference: 74110 uMol/L) and urea within normal limits. Thyroid function tests were unremarkable. Elevated HbA1c at 9.8% (reference: 4.5-6.4%) suggested poor glycemic control. The cholinesterase level was within the normal range, reducing the likelihood of organophosphate poisoning. The peripheral blood smear (PBS) showed normocytic normochromic RBCs with no atypical cells. The findings, including liver dysfunction, abnormal coagulation, and possible inflammatory response, suggested acute liver injury.

The patient showed improvement in acidosis. She was in stable condition at the time of discharge after a 7-day hospital stay and was prescribed fluoxetine 20 mg once daily for depression.

## DISCUSSION

Cartap is a thiocarbamate pesticide which is mostly available in two forms: 1) 4% GR (granules) used for controlling paddy and sugarcane pests, and 2) cartap hydrochloride 50% SP (soluble powder) used for controlling diamond black moth in cabbage and cauliflower.^3,4^ WHO classifies cartap as class II toxicity, labelling it as a mildly hazardous specialised product, while the Insecticide Resistance Action Committee (IRAC) classifies it as a Class 4 insecticide which is considered relatively safe and non-toxic to humans.. Despite being labelled as mildly hazardous, fatalities have been reported. Oral LD50 in the monkey is 100200 mg/kg body weight, which is high compared to other pesticides.^1^

Cartap toxicity can occur through various routes, including ingestion, skin contact, and eye exposure.^9^ The molecule blocks the neuromuscular junction, inhibiting the postsynaptic nicotinic acetylcholine receptors. This inhibition can lead to symptoms such as increased salivation, nausea, vomiting, and abdominal pain, with occasional tremors in the arms and legs often misdiagnosed and treated as OP poisoning.^6^ Cartap also causes the release of calcium from the sarcoplasmic reticulum that can lead to myogenic contracture and injury to the diaphragm, leading to deaths in animal models.^4^ In severe cases, the patient may experience convulsions, respiratory failure, and potentially death. Mechanical ventilation may be required for patients with such cases, which in most cases are extubated within 48 hours.^4,7,8^

Cartap has also shown an increase in reactive oxygen species level, which is the basis of antioxidants like Vitamins C and E, catalase, superoxide dismutase, and N-acetyl cysteine, which can potentially inhibit the harmful effects caused by cartap. The toxicity of cartap is increased when taken together with drugs, food, alcohol, or other substances that inhibit cytochrome P450.^6^ In our patient, the co-ingestion of alcohol may have potentiated the neuromuscular toxicity[3]. Mydriasis has been reported as an ocular manifestation in ocular manifestations, but in our case, nystagmus was seen.^3^

Due to the rarity of poisoning, there are no clear guidelines, and immediate decontamination followed by symptomatic treatment stays the main treatment strategy. It has been shown that sodium dimercaptopropane sulfonate (DMPS) and sodium dimercaptosuccinate (DMS) can completely reverse the effects of these compounds on animal models that slow down breathing.^2^ The currently recommended antidotes for Cartap poisoning are 100-200 mg of L-cysteine administered intravenously or 20-60 mg of British Anti-Lewisite (dimercaprol; 2,3-dimercaptopropanol) given intramuscularly. While BAL has been effective in many cases, its limited availability often leads to using NAC, which has also shown positive outcomes in numerous instances.^2^ Kiyota et al. reported a similar case of Cartap poisoning, where prompt gastric lavage and conservative management led to quick recovery, as seen in our case.^9^ So the ultimate reason for the better outcome in our patient was immediate decontamination with soap water lavage and prompt ventilation.

## CONCLUSIONS

The agricultural sector must raise awareness about the risks of pesticides like cartap, and research into their long-term effects will shape safety standards. Promoting alternative pest control methods and sustainable farming practices can reduce reliance on harmful pesticides, protecting human health and the environment.
